# iNetModels 2.0: an interactive visualization and database of multi-omics data

**DOI:** 10.1093/nar/gkab254

**Published:** 2021-04-13

**Authors:** Muhammad Arif, Cheng Zhang, Xiangyu Li, Cem Güngör, Buğra Çakmak, Metin Arslantürk, Abdellah Tebani, Berkay Özcan, Oğuzhan Subaş, Wenyu Zhou, Brian Piening, Hasan Turkez, Linn Fagerberg, Nathan Price, Leroy Hood, Michael Snyder, Jens Nielsen, Mathias Uhlen, Adil Mardinoglu

**Affiliations:** Science for Life Laboratory, KTH – Royal Institute of Technology, Stockholm SE-171 21, Sweden; Science for Life Laboratory, KTH – Royal Institute of Technology, Stockholm SE-171 21, Sweden; School of Pharmaceutical Sciences & Key Laboratory of Advanced Drug Preparation Technologies, Ministry of Education, Zhengzhou University, Zhengzhou, Henan Province, PR 450001, China; Science for Life Laboratory, KTH – Royal Institute of Technology, Stockholm SE-171 21, Sweden; Bash Biotech Inc, 600 West Broadway, Suite 700, San Diego, CA, USA; Bash Biotech Inc, 600 West Broadway, Suite 700, San Diego, CA, USA; Bash Biotech Inc, 600 West Broadway, Suite 700, San Diego, CA, USA; Department of Metabolic Biochemistry, Rouen University Hospital, 76000 Rouen, France; Normandie Univ, UNIROUEN, CHU Rouen, INSERM U1245, 76000 Rouen, France; Bash Biotech Inc, 600 West Broadway, Suite 700, San Diego, CA, USA; Bash Biotech Inc, 600 West Broadway, Suite 700, San Diego, CA, USA; Department of Genetics, Stanford University, Stanford, CA 94305, USA; Providence Cancer Center, Oregon Area, Portland, OR, USA; Department of Medical Biology, Faculty of Medicine, Atatürk University, Erzurum, Turkey; Science for Life Laboratory, KTH – Royal Institute of Technology, Stockholm SE-171 21, Sweden; Institute of Systems Biology, Seattle, USA; Institute of Systems Biology, Seattle, USA; Department of Genetics, Stanford University, Stanford, CA 94305, USA; Department of Biology and Biological Engineering, Chalmers University of Technology, Gothenburg, Sweden; Science for Life Laboratory, KTH – Royal Institute of Technology, Stockholm SE-171 21, Sweden; Science for Life Laboratory, KTH – Royal Institute of Technology, Stockholm SE-171 21, Sweden; Centre for Host–Microbiome Interactions, Faculty of Dentistry, Oral & Craniofacial Sciences, King's College London, London SE1 9RT, UK

## Abstract

It is essential to reveal the associations between various omics data for a comprehensive understanding of the altered biological process in human wellness and disease. To date, very few studies have focused on collecting and exhibiting multi-omics associations in a single database. Here, we present iNetModels, an interactive database and visualization platform of Multi-Omics Biological Networks (MOBNs). This platform describes the associations between the clinical chemistry, anthropometric parameters, plasma proteomics, plasma metabolomics, as well as metagenomics for oral and gut microbiome obtained from the same individuals. Moreover, iNetModels includes tissue- and cancer-specific Gene Co-expression Networks (GCNs) for exploring the connections between the specific genes. This platform allows the user to interactively explore a single feature's association with other omics data and customize its particular context (e.g. male/female specific). The users can also register their data for sharing and visualization of the MOBNs and GCNs. Moreover, iNetModels allows users who do not have a bioinformatics background to facilitate human wellness and disease research. iNetModels can be accessed freely at https://inetmodels.com without any limitation.

## Graphical Abstract

**Graphical Abstract:**
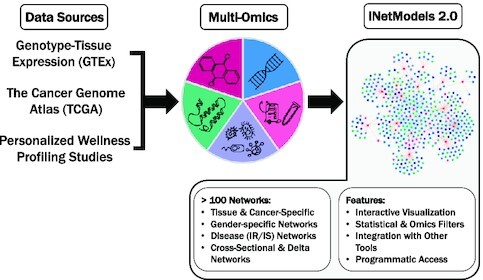
iNetModels 2.0: an interactive visualization and database of multi-omics data.

## INTRODUCTION

During the past decade, the development of high-throughput technologies has dramatically decreased the cost of generating large-scale multi-omics datasets (1). This has opened up the possibilities to study human wellness and diseases systematically (2). Although analysis of individual omics methodologies has been proven beneficial in different clinical applications, integrating multi-omics data may offer novel insights and provide a more comprehensive understanding of biological functions in the human body in health and disease (3). For instance, a recent study integrated time series phenomics, metabolomics and fluxomics data from the subjects with various degrees of liver fat and revealed that non-alcoholic fatty liver disease (NAFLD) is associated with glycine and serine deficiency (4). Another longitudinal phenomics, transcriptomics, metagenomics and metabolomics data have been generated for 10 subjects during a two-week follow-up study. This study has illustrated the rapid metabolic benefits of an isocaloric carbohydrate-restricted diet on NAFLD patients and revealed the molecular mechanisms associated with the metabolic changes (5). Moreover, several other studies have also demonstrated the benefit of performing longitudinal multi-omics data analysis in systematically capturing human diseases' dynamics (6–8).

**Figure 1.**
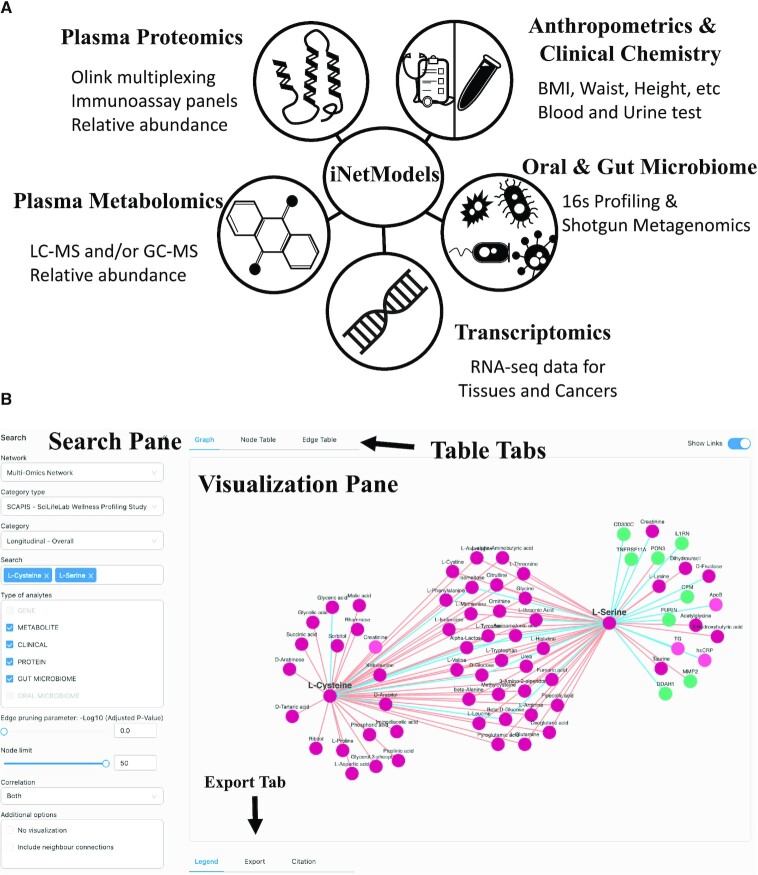
(**A**) Summary of multi-omics data presented in iNetModels (**B**) Working page of iNetModels.

To provide a better framework for facilitating these types of investigations, we created iNetModels. This user-friendly platform provides exploratory capabilities and interactive and intuitive visualization of clinical chemistry, anthropometric parameters, plasma proteins, plasma metabolites, oral microbiome and gut microbiome associated with the user-queried features (Figure 1A). The data in iNetModels are obtained from recent studies, where large-scale Multi-Omics Biological Networks (MOBNs) analyses have been performed for individuals with different metabolic conditions. Moreover, we retrieved data from The Genotype-Tissue Expression (GTEx) Project and The Cancer Genome Atlas (TCGA), created normal tissue- and cancer-specific Gene Co-expression Networks (GCNs) and presented the networks in the iNetModels (Figure 1A). The user can simultaneously query for 1–5 features, visualize the selected features and their neighbouring features, download the associated network in both table and figure format, and analyse them using independent network analysis tools, including Cytoscape (9) and iGraph (10). We also encourage users to upload their networks into iNetModels and make those networks accessible to a broader audience for creating an open platform to share and visualize their networks. To our knowledge, iNetModels is the first database that provides associations between the multi-omics data obtained from the same individuals in a physiological context rather than using a text mining method.

## PLATFORM DESCRIPTION AND FEATURES

iNetModels 2.0 is a web-based platform that includes two main features: a database and an interactive visualization of multi-omics network analysis (Figure 1B). It is an updated and improved version of the previous work, TCSBN, released in 2017 (11). First, we reconstructed the GCNs presented in TCSBN based on more recent datasets and expanded it to 85 different tissue- and cancer-specific networks. Second, we broadened the platform by adding 20 MOBNs from multiple independent studies. Finally, we improved the backend and frontend of the platform for a better user experience. To our knowledge, this platform is the only publicly available platform that enables the exploration of MOBNs that were generated based on personalized omics data.

### Data sources

The tissue- and cancer-specific GCNs were constructed using GTEx (v8) and TCGA (v27.0-fix) primary tumour data, respectively. We retrieved the gene transcripts per million (TPM) from GTEx Portal and the fragment per kilobase of transcript per million mapped reads (FPKM) files of the TCGA program from the Genomics Data Commons (GDC) portal.

MOBNs were generated as the consensus of clinical variables, plasma proteomics, plasma metabolomics and metagenomics data based on from three independent longitudinal wellness profiling studies: (i) SCAPIS-SciLifeLab Wellness Profiling study (12): clinical variables, metabolomics, proteomics and gut metagenomics data from 4 visits over a year from 101 individuals (50–65 years old), (ii) P100 study (13): clinical variables, metabolomics and proteomics data collected from three visits of 108 individuals (21–89+ years old) in over 9 months, (iii) Integrative Personal Omics (14): clinical variables, metabolomics and proteomics data from three visits over weight gain and loss period (97 days) from 10 insulin-sensitive (IS) and 13 insulin-resistance (IR) overweight individuals. Moreover, we generated MOBNs for two different clinical trials where combined metabolic activators (CMA) administered to NAFLD and COVID-19 patients: (i) NAFLD CMA administration: clinical variables, metabolomics, proteomics and metagenomics data for the oral and gut microbiome of 31 NAFLD patients from three visits over 70 days, and (ii) COVID-19 CMA administration: Clinical variables, metabolomics and proteomics data collected 93 COVID-19 patients from two visits over 14 days.

### Network generations

Each dataset was pre-processed independently before the network generation. The gene expression data were filtered to remove lowly expressed genes (TPM < 1 or FPKM < 1) in each tissue or cancer type to avoid bias. For the multi-omics data, we corrected them by removing the effects of age (in all networks) and sex (in the non-gender-specific networks) using trimmed mean robust regression (13). We combined the data from each omics into a matrix to generate a network and analysed it using the Spearman correlation function from the SciPy package. Each data source was processed independently. After filtering for striking correlations between the pairs, we filtered the pairs with FDR <0.05. Furthermore, we performed community detection analysis using the Leiden algorithm (15) in the iGraph package to identify sub-network clusters for downstream analysis.

Moreover, in the longitudinal wellness profiling studies, we calculated both cross-sectional and delta networks. These networks are generated based on the methodology presented in the P100 wellness study (13). Cross-sectional networks were calculated by correlating data from all visits to represent correlations in the context of individualized variation. Meanwhile, delta networks were calculated by correlating the analyte changes between visits to allow users to investigate features that co-vary within the same time intervals.

The number of the nodes and edges in the generated networks varying between 5673 (liver cancer)—12 581 (testis) nodes and 63 292 (endocervix)—132 223 286 (testis) edges in GCNs, whereas 279 (Delta IS)—1042 (NAFLD CMAs) nodes and 1034 (Delta IS)—566 390 (SCAPIS—SciLifeLab Longitudinal Male) edges in MOBNs (Supplementary Table S1).

### Features

The iNetModels platform provides users with a vast number of pre-computed biological networks. Users may choose the suitable networks from tissue- or cancer-specific GCNs to gender or IR/IS-specific MOBNs based on their study's focus. To search within a specific network (Figure 1B, Supplementary Figure S1), firstly, users need to select the specific network category (GCNs or MOBNs), then select the specific network type (normal tissue, cancer, or multi-omics study), and subsequently select the specific network. Following that, users need to input the commonly known names of analytes (gene, protein, metabolite name etc.) of interests using the free text and/or drop-list. Optionally, users can filter the network based on the analyte types (in multi-omics networks) and statistical properties, e.g. FDR and Spearman correlation ranking (positive, negative, or both correlations). In the web interface, the number of neighbouring nodes is limited to a maximum of 50 neighbours per-queried nodes to avoid browser unresponsiveness. Furthermore, in the ‘additional options’ box, users can choose to include neighbours' connections in the networks and disable network visualization when only network tables are needed.

Once the network is generated, the users can interactively explore the network, download the network as a figure and its content in a table format for further exploration and downstream analysis. All information about each analyte (network nodes) and related associations (network edges) is shown in the table area: the analyte name, short description, unit, correlation and the *P*-value of the significant associations etc. Besides, wherever possible, analytes are linked with external databases such as KEGG (16), Human Protein Atlas (17–19), Uniprot (20) and HMDB (21) to facilitate further biological interpretation and investigation. All of this information can be downloaded directly and is compatible with other network analysis tools and software, such as Cytoscape or iGraph package in Python and R. By using these tools, users can merge multiple networks and perform additional downstream analysis.

Moreover, in the iNetModels 2.0, we implemented programmatic access to the database using an in-house-built Python package that can be found under the ‘API’ section. With the API, users can retrieve more extensive networks (>50 neighbours per-queried nodes) programmatically. We currently limit the query to one query per second.

### Case study related to administration of CMA in NAFLD

One of our platform's unique features and superior strengths is that iNetModels 2.0 is the first and only platform supporting the exploration of MOBNs based on personalized data. This is a great advantage to avoid bias since all data were analysed in a paired manner.

**Figure 2.**
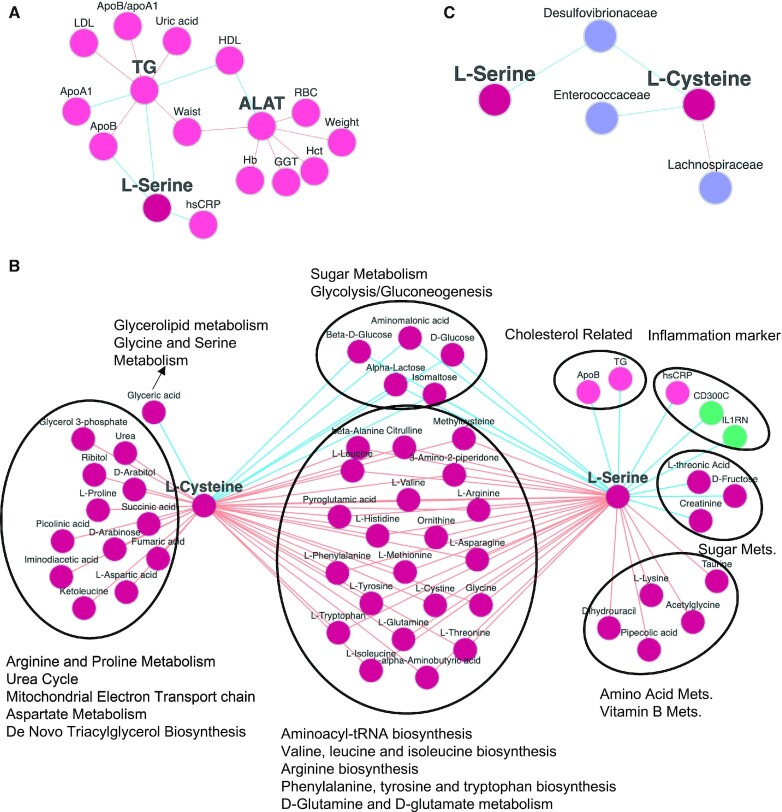
(**A**) Validation of the hypothesis about the supplementation of L-Serine that was associated with the decrease in the plasma triglycerides levels and liver enzyme (ALAT) in the SCAPIS-SciLifeLab Wellness Profiling study. (LINK). (**B**) The two main components of the supplementation (l-cysteine and l-serine) and their neighbouring analytes are presented based on multi-omics biological networks analysis (LINK). (**C**) The two main components of the supplementation (l-cysteine and l-serine) and their associations with the species in the gut microbiome are presented (LINK). All networks in this figure were taken from the cross-sectional overall SCAPIS-SciLifeLab Wellness Profiling study.

In our recent study (22), we tested a potential therapeutic strategy for NAFLD patients through CMA administration. We provided CMA to 10 subjects involved in the trial and collected plasma samples during the day to generated proteomics and metabolomics data. The data generated in the clinical trial were analysed using metabolic modelling. The results of the analysis were validated by performing animal experiments, where L-serine was supplemented to mouse and a reduction in the liver triglycerides (TG) and markers of liver tissue functions, e.g. ALAT, ASAT, and ALP was observed. We validated these results in two independent MOBNs in iNetModels 2.0 (Figure 2A, Supplementary Figure S2A). Our analysis revealed that two metabolic activators, including l-serine and l-cysteine are positively associated with metabolites related to branched amino-acid metabolism (i.e. l-valine, l-isoleucine and l-leucine) and negatively associated with plasma glucose level, supporting the main findings of the study (Figure 2B). The MOBNs also showed that l-serine is associated negatively with cholesterol-related clinical variables (ApoB, TG) and several other inflammation markers (hsCRP, IL1RN and CD300C).

Based on the same network, we can filter to show only specific analyte types (Figure 2C, Supplementary Figure S2B–D). For example, we used the same network to show the association of l-cysteine and l-serine with only the gut microbiome (Figure 2C), as dysbiosis in the gut microbiome has been associated with NAFLD (23,24). We observed that l-cysteine had a positive correlation with the abundance of Lachnospiraceae and a negative correlation with the abundance of the Enterococcaceae family. Independent studies have shown that the abundance of Lachnospiraceae was increased in cirrhosis (25,26), whereas the abundance of Enterococcaceae was decreased in cirrhosis (25). We also found that the levels of both l-serine and l-cysteine were negatively correlated with the abundance of the Desulfovibrionaceae family, which was increased with the severity of NAFLD (27).

## CONCLUSION

iNetModels is a unique platform that gathers a broad spectrum of biological networks, from tissue- and cancer-specific GCNs to MOBNs based on personalized data. This platform may help researchers to perform exploration and validation experiments, identify functional relationships between the analytes, and most importantly, provide new insights into the biological experiments and ultimately identify potential drug targets and biomarkers. The case study about the supplementation of CMA in NAFLD patients has shown the efficient usage of this platform in testing and validating hypotheses as well as confirming results from experiments or clinical trials. This platform is designed in a user-friendly way and it is freely accessible to a wide range of users, including bench scientists with limited or no formal bioinformatics background. We also envisage that this platform will be a key resource for computational biologists working in omics data integration, network science, systems biology and systems medicine. In this context, we expect iNetModels to be an essential resource for more in-depth multi-omics analysis that may reveal novel molecular mechanisms underlying human wellness and disease.

In the future, in addition to the inclusion of more studies, we are planning to expand the functionalities of the platform. First, with the increasing number of personalized wellness profiling study, we plan to develop a method to build a consensus MOBN based on various studies to increase the robustness of the findings generated from this platform. Second, we plan to add a functional analysis feature to show the enriched pathways or biological processes by integrating the selected network information to other databases, such as KEGG (16) or Metabolic Atlas (28). Finally, we will add integration with users’ quantitative omics data or statistical inference results to identify the significantly altered nodes in various perturbations.

## DATA AVAILABILITY

iNetModels can be accessed freely by everyone at https://inetmodels.com without any limitation. All codes used to generate the network and the network data are available under the ‘API’ and ‘Help’ section of the website.

## Supplementary Material

gkab254_Supplemental_Files
