# Supplementary material for: iNetModels 2.0: an interactive visualization and database of multi-omics data
Source: Nucleic Acids Res. 2021 Apr 13;49(W1):W271–6. doi: 10.1093/nar/gkab254 (PMC8262747; doi:10.1093/nar/gkab254)
Supplement: gkab254_Supplemental_Files [file gkab254_supplemental_files.zip › Supplementary Figures.pdf]

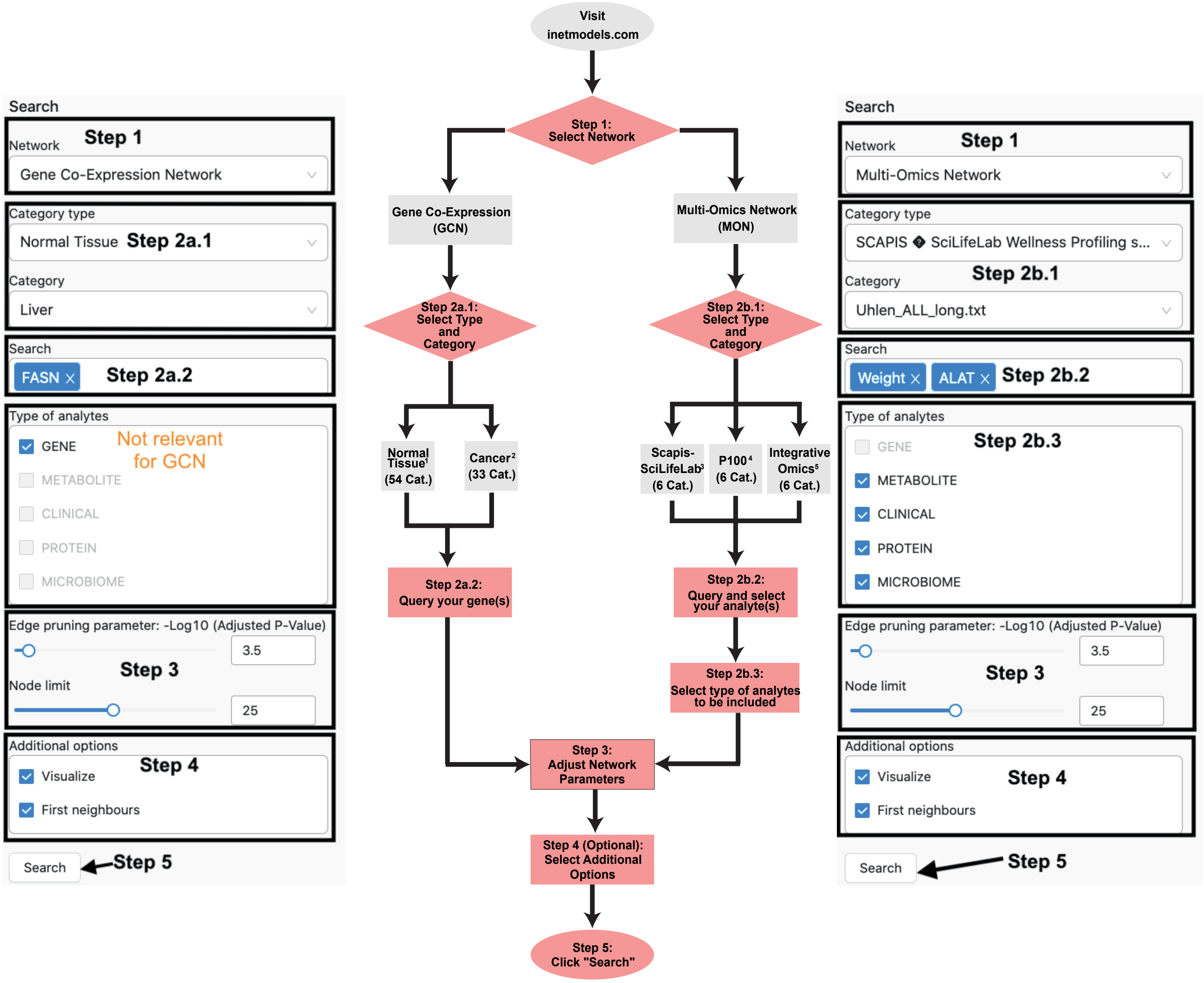

Supplementary Figures 1 User query flow in iNetModels to search for specific analytes of interest.

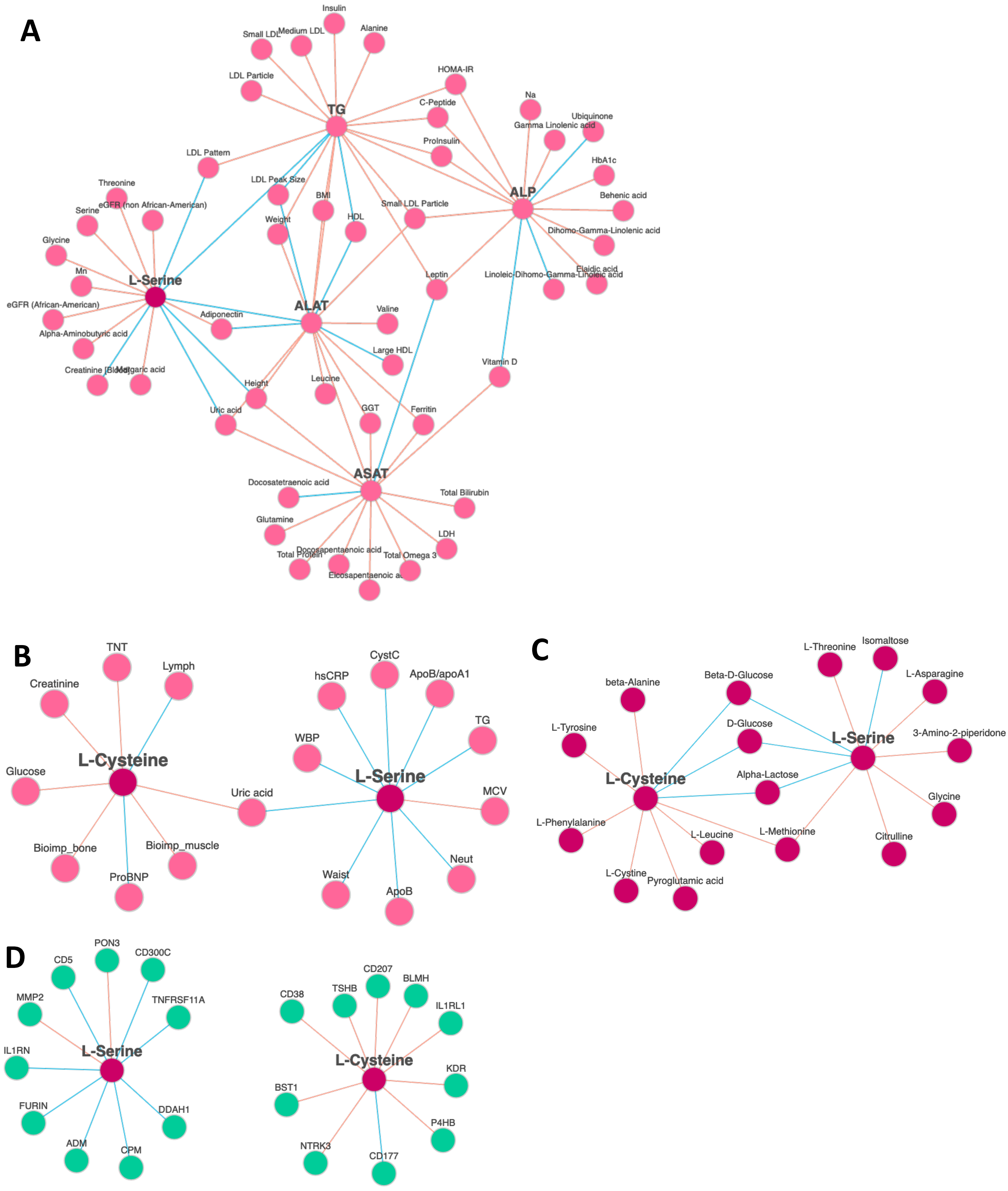

Supplementary Figures 2 (A) Validation of the hypothesis about the supplementation of L-Serine that was associated with the decrease in the plasma triglyceride levels and liver enzymes (ALAT, ASAT, and ALP) in the P100 Study. (B) Clinical data (LINK), (C) Metabolites (LINK), and (D) Proteins (LINK) associated with the two main components of the supplementation (L-Cysteine and L-Serine) based on multi-omics biological networks analysis in the SCAPIS-SciLifeLab Wellness Profiling Study.
